# Development and validation of a droplet digital PCR method for quantifying lentiviral vector infectious titer

**DOI:** 10.1016/j.heliyon.2024.e38512

**Published:** 2024-10-01

**Authors:** Xueling Wu, Xiaoya Zhou, Yueming Wang, Jian Wu, Qian Liang, Xu Yang, Kehua Zhang, Shufang Meng

**Affiliations:** a National Institutes for Food and Drug Control, Beijing, 100050, China; b State Key Laboratory of Drug Regulatory Science, Beijing, 100050, China; c Beijing Minhai Biotechnology Co., Ltd., Beijing, 102600, China

**Keywords:** Lentiviral vectors, Infectious titer, Digital droplet polymerase chain reaction (ddPCR), Digestive enzymes, Method validation

## Abstract

Lentiviruses, with their high transduction efficiency and gene expression levels, are widely used as gene delivery vectors in the development of chimeric antigen receptor T cells (CAR-T) and other genetically modified cell therapies. Accurate determination of the lentiviral vector infectious titer is essential to ensure effective transduction and product consistency. In this study, we developed an efficient method for lentiviral vector titration based on digital droplet polymerase chain reaction (ddPCR) technology, enabling absolute quantification of the target gene. Benzonase treatment of non-transduced plasmids substantially shortened the experimental period, reducing cell culture duration from 10-14 days–3 days. The method was rigorously validated by assessing specificity, working range, limit of quantification, precision, accuracy, and robustness. This study demonstrates the feasibility of combining enzymatic digestion with ddPCR to quantify lentiviral vector infectious titer and provides a detailed and readily adaptable methodology for the scientific community.

## 1 Introduction

Lentiviral vectors, members of the *Retroviridae* family, are commonly produced by transfecting HEK293T cells with high concentrations of transfer and packaging plasmids [1]. These vectors are widely used for gene delivery due to their ability to infect both dividing and non-dividing cells, and to stably integrate into the host cell genome [2]. For example, transgenes encoding a chimeric antigen receptor (CAR) and the T cell receptor (TCR) are usually introduced into T cell genomes by non-replicating lentiviral vectors [3]. In the production of cell therapy products, accurate determination of the functional titer of lentiviral vectors is crucial for successful production and product consistency. Moreover, for lentiviral drugs, infectious titer quantification is crucial when assessing therapeutic dosing to reduce the risk of low efficacy or toxic side effects, thereby improving treatment outcomes [4].

The viral vector infectious titer is defined as the number of viruses that have successfully transduced and integrated into the cell genome per unit volume. Various methods have been used to measure the infectious titer of lentiviral vectors; fluorescence-activated cell sorting (FACS) and real-time quantitative PCR (qPCR) are most commonly adopted. However, both methods have limitations. FACS offers a rapid, direct approach to detecting lentiviral titers. However, this method has requires specific antibodies and cannot differentiate between cells infected with a single vector and cells infected with multiple vectors. Furthermore, FACS cannot detect integrated but non-functional viral vector copies that do not lead to transgene expression [5,6], potentially leading to underestimation of the viral vector titer. qPCR is the most accurate method for measuring lentiviral vector titers, which it achieves by quantifying the copy number of proviral DNA within each host genome. However, qPCR is time-consuming, and accurate quantification requires the preparation of a standard curve using a control template with a known copy number [1,5].

In addition to FACS and qPCR methods, droplet digital PCR (ddPCR) has also been established for lentiviral vector titer quantification [7]. ddPCR is a technology used for detection and quantification of specific nucleic acid sequences, which encapsulates DNA samples into tens of thousands of tiny droplets [8]. Each droplet generated in ddPCR serves as an individual reaction chamber for product amplification and is analyzed separately after the reaction to quantify two or more fluorescent target molecules. When this principle is applied to a virus titration experiment, the average copy number of lentiviral vectors in the cell population is calculated using a Poisson distribution algorithm [3]. The ddPCR method established by Wang et al. for quantifying lentiviral vector titer demonstrates high similarity with FACS and qPCR, and it offers the following advantages over these two methods: First, by partitioning amplification inhibitors into different sub-reactions, ddPCR exhibits increased resistance to inhibitors and provides enhanced stability [9]. Second, the ddPCR method demonstrates high sensitivity in viral nucleic acid detection, despite the high background concentration of cellular genomic DNA [10]. Third, the copy number concentration of the target sequence is determined independently of a calibration curve, simplifying the assay and making its results more objective [7,8,11]. However, unincorporated lentiviral elements must be diluted before genomic DNA extraction for ddPCR analysis; this dilution requires subculturing for 10–14 days, which is time-consuming. In this study, we established an improved ddPCR method for the quantification of lentiviral vector infectious titer by targeting WPRE and β-globin; our method exhibits good specificity, repeatability, accuracy, and robustness. This method uses HT1080 as the infected cell line and uses enzymatic treatment to eliminate the effects of residual plasmids on the detection results, thereby shortening the culture time to 3 days post-infection and greatly improving detection efficiency. These findings provide insights concerning the application of ddPCR in lentiviral vector titer assessment and offer a new option for quality control of cell therapy products.

## 2 Results

### 2.1 Method development

#### 2.1.1 Cell line selection

**Fig. 1:**
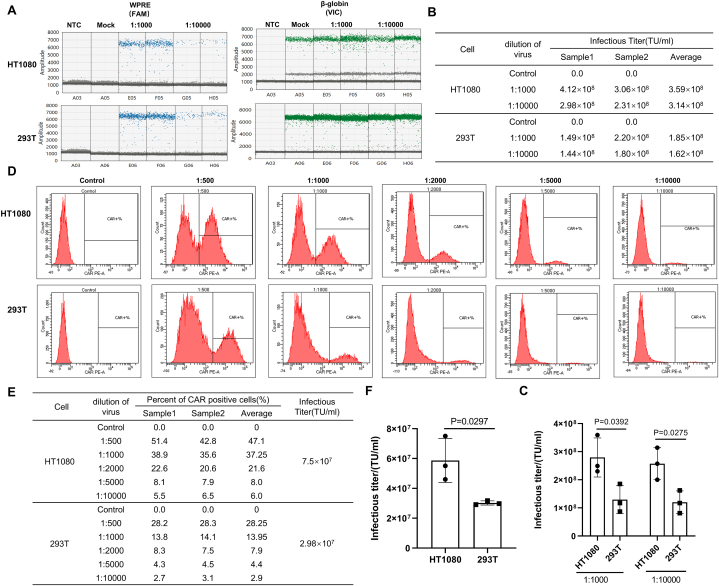
Selection of cell line for lentiviral infection.

To select a suitable cell line for quantifying lentiviral titer, we tested the lentiviral vector sensitivities of HT1080 and 293T cells; we also explored the degree of difficulty when these cells were used for experimental procedures. Both cell types can be infected by lentiviral vectors and yield infectious titers, but the transduction titers measured were twofold higher in HT1080 cells than in 293T cells at virus dilutions of 1:1000 and 1:10000 (Fig. 1A–C). Additionally, we used FACS to evaluate the lentiviral vector sensitivities of the two cell types. Consistent with the ddPCR results, HT1080 cells exhibited a twofold higher infectious titer value compared with 293T cells (Fig. 1D–F). Therefore, HT1080 cells demonstrated higher susceptibility to lentiviral vectors and offered advantages in the detection of low titer samples. Furthermore, due to the adherent nature of 293T cells, they were likely to become detached during harvesting, especially during prolonged culture, which could affect the accuracy of the results. Based on these factors, we chose the HT1080 cell line for further experiments.

(A–C) Infectious titers were measured in HT1080 and 293T cells using the ddPCR method. (A) Positive droplets（blue or green）and negative droplets（grey）detected by ddPCR. (B) The virus number per genome was calculated based on measurement of *WPRE* and β-globin copies. (C) P-values were determined by two-sample *t-tests* based on three independent experiments. The difference between HT1080 cells and 293T cells was statistically significant (P < 0.05). (D–F) Lentiviral vector infection of HT1080 cells and 293T cells and quantification of infectious titer by FACS. (D) Lentiviral vector stock was serially diluted before cell infection experiments. Three days after infection, the percentage of cells expressing CAR was determined by FACS. (E) Percentages of CAR-positive cells in two duplicate wells infected by lentiviral vector at the indicated dilutions. (F) P-values were determined by two-sample *t-tests* based on three independent FACS experiments. The difference between HT1080 cells and 293T cells was statistically significant (P < 0.05).

#### 2.1.2 Target gene selection

**Fig. 2:**
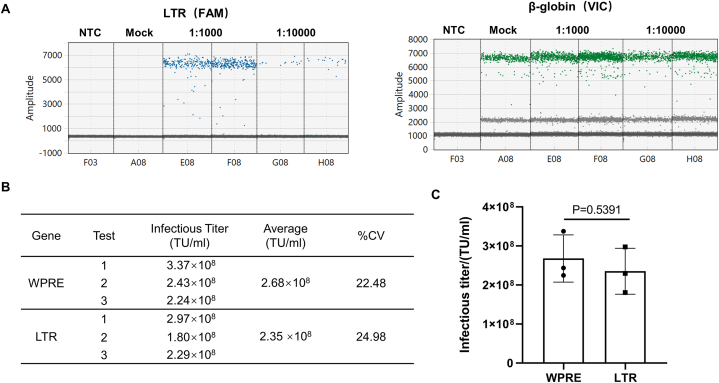
Target gene selection (A) Positive and negative droplets detected by ddPCR targeting *LTR*. (B) Infectious titer was calculated based on measured copy numbers for *WPRE* or *LTR* and *β-globin*. (C) P-values were determined by two-sample *t-tests* based on three independent experiments. The difference between *LTR* and *WPRE* was not statistically significant (P > 0.05).

In this section, we aimed to select target genes for further analysis. The lentivirus long terminal repeat (*LTR*) and woodchuck hepatitis virus post-transcriptional regulatory element (*WPRE*) were chosen as candidate genes. The *LTR* sequences are highly conserved, making them stable and reliable targets for measurement using ddPCR. *WPRE*, a post-viral transcriptional regulatory element, has demonstrated reliability as a target in qPCR assays to determine infectious titer [12]. We compared ddPCR results for *WPRE* with results for the commonly used *LTR* (Fig. 2). Droplet separation was clear for both target genes (*LTR* and *WPRE*) (Fig. 2A). Based on these results, both genes were suitable for our tests. Considering the accessibility of primer and probe sequences, *WPRE* was selected as the target gene for subsequent experiments.

#### 2.1.3 Selection of digestive enzymes

**Fig. 3:**
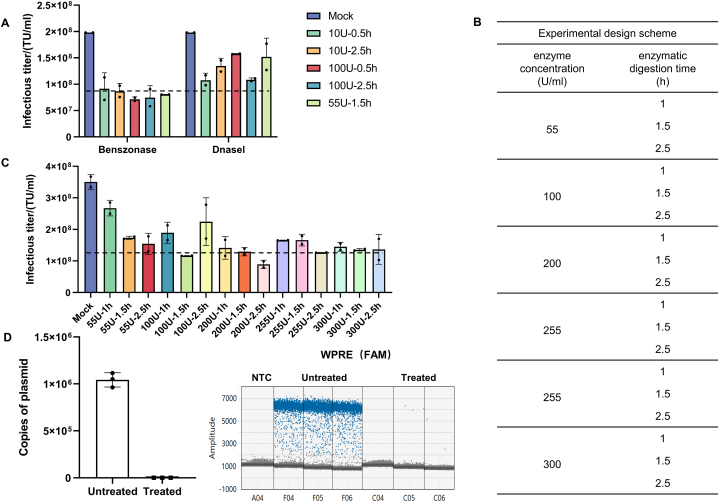
Selection of digestive enzymes and conditions. (A) Infectious titers after enzyme treatment. Compared with the untreated group, the Benzonase and DNase I treatments both showed a significant decrease in infectious titer; the Benzonase treatment demonstrated a more pronounced decrease. (B) Experimental design for selecting the enzyme concentration and enzymatic digestion interval. (C) Infectious titers were determined by enzyme treatment under different conditions. (D) Plasmid copy numbers in enzyme-treated and untreated samples. Transfer plasmids (1E6 copies) were added to HT1080 cells and digested with 100 U/ml Benzonase for 1.5 h. Benzonase treatment reduced the plasmid copy number to zero. NTC: no template control.

To assess the effect of enzyme treatment on unincorporated lentiviral elements and determine which enzyme is more suitable for quantifying viral titer, we tested Benzonase, which can digest extracellular free DNA and RNA, and DNase I, which can only digest extracellular free DNA. Compared with the untreated group, infectious titers significantly decreased after enzyme treatment, indicating the importance of this step. These results demonstrated that under the same conditions, the plasmid was more thoroughly digested by Benzonase than by DNase I (Fig. 3A). Therefore, Benzonase was chosen for subsequent studies.

#### 2.1.4 Selection of enzyme concentration and enzymatic digestion interval

To determine the optimal enzymatic reaction conditions, we tested various combinations of enzyme concentrations and digestion intervals (Fig. 3B). The results showed that Benzonase concentrations ranging from 100 to 300 U/ml and digestion intervals ranging from 1 to 2.5 h achieved acceptable digestion (Fig. 3C). To balance cost-effectiveness and efficient digestion, we chose 100 U/ml Benzonase and 1.5 h of digestion for further experiments.

To demonstrate thorough plasmid digestion by Benzonase, we added 1E6 copies of a transfer plasmid containing the *WPRE* element to HT1080 cells and performed digestion with 100 U/ml Benzonase for 1.5 h. The amount of plasmid added was consistent with the maximum residual amount of plasmid in the lentiviral vector preparation. Genomic DNA was extracted from the Benzonase-treated and untreated groups and simultaneously subjected to ddPCR. The results showed *WRPE* negativity in the Benzonase-treated group and positivity in the untreated group (Fig. 3D). These findings indicate that residual plasmids can be completely digested under the enzyme treatment conditions established in this study.

#### 2.1.5 The protocol of ddPCR method for quantifying lentiviral vector infectious titer

Experimental design: Prepare one or more viral vector dilutions to infect cells. Each dilution should be used to infect two wells. Concurrently, include a "Mock" well without viral vector as a negative control. In the ddPCR assay, a positive control should be included, such as the genome of a cell line with a known *WPRE* copy number as the ddPCR template. During analysis of the results, the negative control group should have a vector copy number (VCN) of zero; the VCN accuracy for the positive control group should be ≥ 85 %. At least one dilution in the experimental group should be within the working range of the method, and the CV of the infectious titer derived from replicates should be ≤ 25 %, and the infectious titer is calculated as the average titer of replicates. If multiple viral vector dilutions fall within the working range, calculate infectious titers for each dilution separately. The final infectious titer is calculated as the average titer across all dilutions. If the same sample is tested in multiple experiments, the CV of the infectious titer from these experiments should be ≤ 25 %. The final infectious titer is calculated as the average of infectious titers from all experiments.

#### 2.1.6 Viral transduction

1. One day before transduction, plate HT1080 cells at a density of 3 × 10^5^ cells per well in a 6-well plate.
2. Twenty-four hours after seeding, count the number of cells in one well using a cell counter.
3. Dilute the viral vector (a 1000-fold dilution is recommended) with complete medium containing polybrene at a final concentration of 8 μg/ml.
4. Add 2 ml of the diluted viral vector to each well, ensuring that each dilution is used to infect two wells. Include a "Mock" well without virus as a control.
5. Centrifuge the plate at 1000×*g* at 30 °C for 30 min, then incubate for 3 h at 37 °C with 5 % CO2.
6. Aspirate the viral diluent and add 2.5 ml of fresh complete medium to each well.
7. Incubate the plate for an additional 72 h before harvesting.

#### 2.1.7 Cell harvesting

8. At the end of the incubation period, aspirate the cell culture supernatant. Collect the cells and wash them with 2 ml of serum-free MEM medium.

#### 2.1.8 Enzyme treatment

9. Add 1 ml of Benzonase enzyme digestion solution (DMEM medium containing 2 mM MgCl_2_ and 100 U/mL Benzonase) to the cell pellet. Incubate the suspension at 37 °C for 1.5 h.
10. After incubation, centrifuge the suspension at 500×*g* for 5 min and discard the supernatant.
11. Wash the cell pellet with 1 ml of phosphate-buffered saline. The pellet can be stored at −20 °C for at least 2 months prior to DNA extraction.

#### 2.1.9 DNA isolation and quantification

12. Extract genomic DNA from the viral vector-transduced cell samples and the untransduced Mock cells (previously treated with Benzonase enzyme) using a High Pure Viral Nucleic Acid Kit (Roche), in accordance with the manufacturer's instructions. Elute the extracted DNA with 100 μl of elution buffer.
13. Measure DNA purity and concentration using a Nanodrop spectrophotometer (Thermo Scientific). The A260/A280 ratio, which indicates protein contamination, should be higher than 1.8.
14. Store the genomic DNA at −20 °C for up to 2 months until ddPCR analysis.

#### 2.1.10 ddPCR copy number assays

15. Dilute the genomic DNA to 20 ng/μl with nuclease-free water for the ddPCR assay.
16. Prepare the PCR mix by combining the following reagents: 1 × ddPCR Supermix for Probes (no dUTP) (Bio-Rad, cat no. 186–3010), 0.9 μM primers and 0.25 μM probes both for *WPRE and β-globin*, 40 ng of cell genomic DNA, and nuclease-free water to adjust the final volume to 20 μl.
17. Use the Bio-Rad AutoDG QX200™ Droplet Generator to automatically generate microdroplets of the PCR mix.
18. Seal the PCR plate containing the microdroplets with pierceable foil using a PX1™ PCR Plate Sealer.
19. Place the sealed PCR plate in a thermocycler and run the following PCR amplification program: 10 min at 95 °C, followed by 40 cycles of 94 °C for 30 s and 60 °C for 1 min, and a final extension at 98 °C for 10 min. Perform all steps using a ramp rate of 2 °C/s.
20. Read the droplets using the QX200™ Droplet Reader.

#### 2.1.11 Data analysis and infectious titer calculation

21. Analyze the data using QuantaSoft software version 1.7.4 (Bio-Rad). Select 0.85 nL as the preferred droplet volume. Exclude samples with fewer than 8000 droplets from the calculation. Calculate infectious titer with dilutions fall within the working range of the method or dilutions with 0.01–1 copies/partition. Ensure that the mean positive droplet amplitude/mean negative droplet amplitude ratio is > 2 for both the target (FAM dye) and the reference (VIC dye) assays.

22. Obtain the copy numbers of *WPRE* and *β-globin* from the QuantaSoft data table. Calculate the VCN was follows: VCN = 2 × *WPRE* copy number/β-globin copy number.
23. Calculate the lentiviral infectious titer using the following formula: infectious titer (TU/ml) = (VCN × number of transduced cells per well)/(virus volume (ml)) × dilution factor. In this formula, the "number of transduced cells" refers to the cell count after 24 h of culture, before addition of the viral vector.

**Fig. 4:**
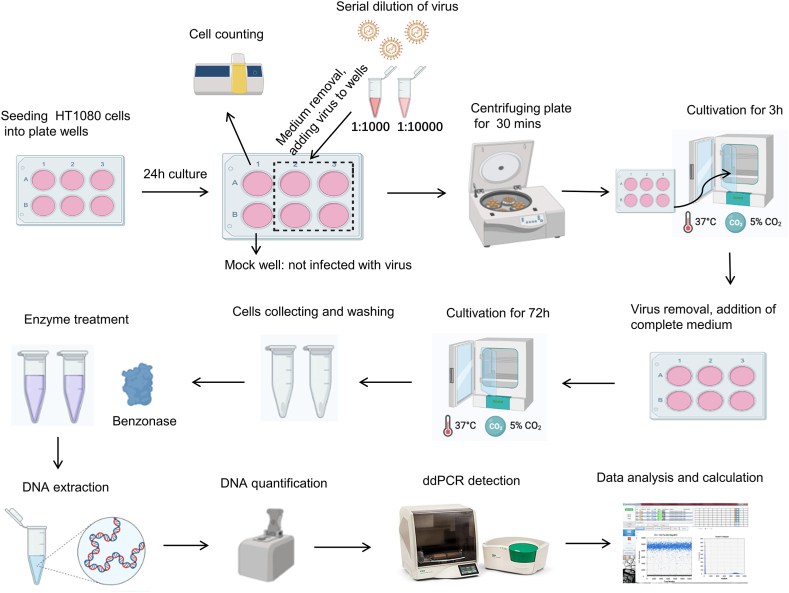
Protocol for quantifying lentiviral vector infectious titer.

The experimental protocol is illustrated in Fig. 4.

### 2.2 Method validation

#### 2.2.1 Specificity

**Fig. 5:**
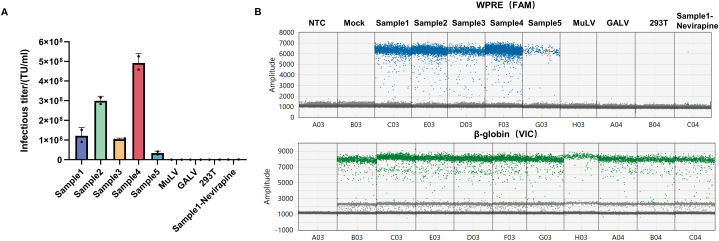
Validation of specificity (A) Infectious titers of samples 1–5, MuLV and GALV virus samples, 293T lysate, and sample 1 in the presence of nevirapine. (B) Droplet distributions of all tested samples.

To verify the specificity of the method, we tested negative samples, including 293T cell lysate and non-related viruses, as well as positive samples of lentiviral vectors (Fig. 5). Lentiviral vectors are usually packaged by 293T cells [13,14], but their lysate may interfere with the quantification of lentiviral vector infectious titer. To address this issue, we co-cultured the 293T lysate with HT1080 cells before genomic DNA extraction. We found that the target gene was not detectable by ddPCR, leading to a negative result. To further verify the effectiveness of this method in excluding interference from non-target substances, samples of known lentiviral vectors (samples 1–5) and non-related viruses (MuLV and GALV) were utilized. The detection results for samples 1–5 were positive, whereas the results for MuLV and GALV samples were negative. To evaluate assay specificity, experiments were conducted with and without nevirapine, a reverse transcriptase inhibitor [[15], [16], [17]]. When nevirapine was added, lentiviral vector (sample 1) transduction was blocked, and the target gene amplification signal was significantly decreased, indicating that this method displays good specificity (Fig. 5). These findings suggest that this method can effectively exclude interference from non-target substances and that it demonstrates good specificity for detecting lentiviral vector DNA.

#### 2.2.2 Working range and limit of quantification (LOQ)

**Fig. 6:**
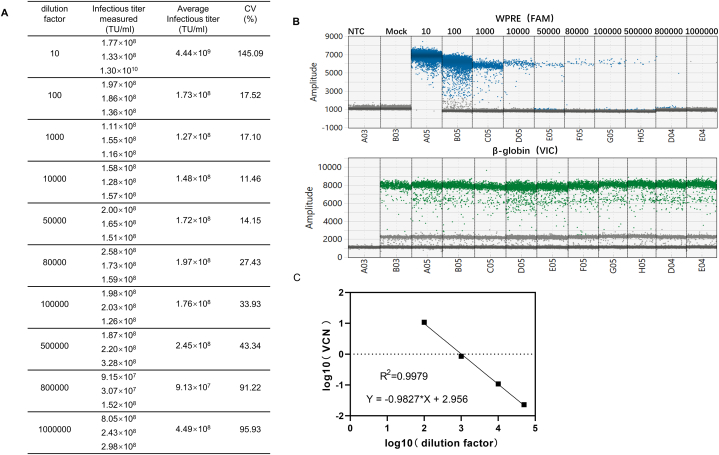
Test results according to dilution ratio (A) Infectious titer andCVs of samples diluted at different ratios. (B) Positive droplet distributions of samples with different dilution ratios. (C) Curve representing the correlation between dilution ratio and VCN.

To test the working range and LOQ, HT1080 cells were transduced with various dilutions of sample 1(Fig. 6A), and genomic DNA was extracted for detection by ddPCR. At a dilution ratio of 10, all droplets showed positive results and the copy number could not be calculated (Fig. 6B), suggesting that the lentiviral vector load was excessively high under these conditions and thus the results were unreliable. When the dilution ratio was greater than 80000, the coefficient of variation (CV) was unacceptable (i.e., >25 %); however, when the dilution ratio was between 100 and 50000, the method displayed an acceptable CV (i.e., <25 %) (Fig. 6A). A strong positive linear correlation was observed between virus copy number (VCN) and dilution ratio, with an R^2^ value of 0.9979 (Fig. 6C). The average value of the four dilutions from 100 to 50000 was 1.55E8, and the CV of these dilution results was 14 %. Therefore, the detectable infectious titer range of this method is approximately 3100 to 1.55E6 transduction Units per mL（TU/ml）. The LOQ of the method was reached at a dilution of 50000 (approximately 3100 TU/ml) with a CV <20 %.

#### 2.2.3 Precision

Precision was evaluated according to intra-assay and inter-assay variation. The results revealed that the CV values in intra-assay and inter-assay measurements were both ≤25 % (Table 1), indicating our method exhibits good repeatability.

**Table 1:**
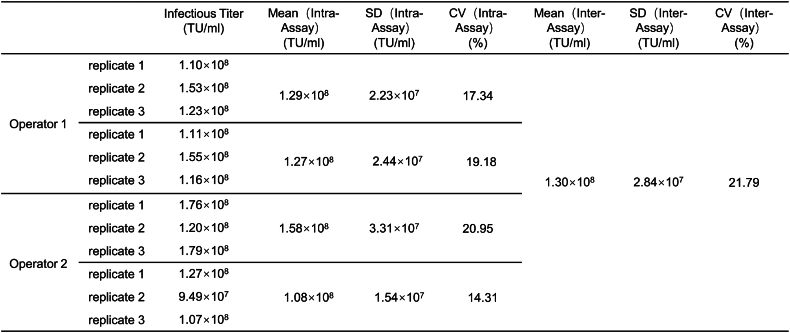
Precisions of infectious titer detection methods.

#### 2.2.4 Accuracy

To examine the accuracy of our ddPCR method, we tested the VCN of Jurkat-CAR cells, which have 13 copies of lentivirus stably integrated into their genome; VCN data for these cells have been validated by integration site detection. Accuracy was calculated as follows: Accuracy = (1− ((∣measured value − true value∣)/true value)) × 100 %. The ddPCR results showed that Jurkat-CAR cells had a VCN of 14.0, with an accuracy >90 % and a CV <5 % based on three independent tests (Table 2). These findings indicated that our ddPCR method displays good accuracy.

**Table 2:**
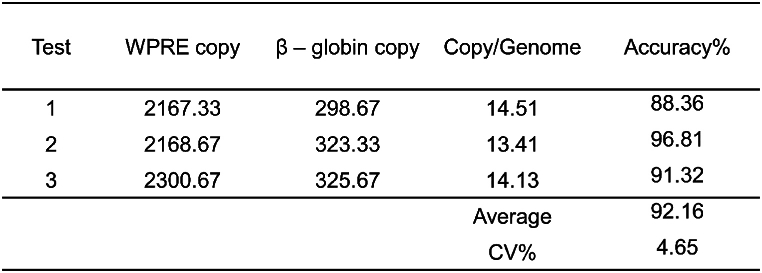
Accuracies of infectious titer detection methods.

#### 2.2.5 Robustness

**Fig. 7:**
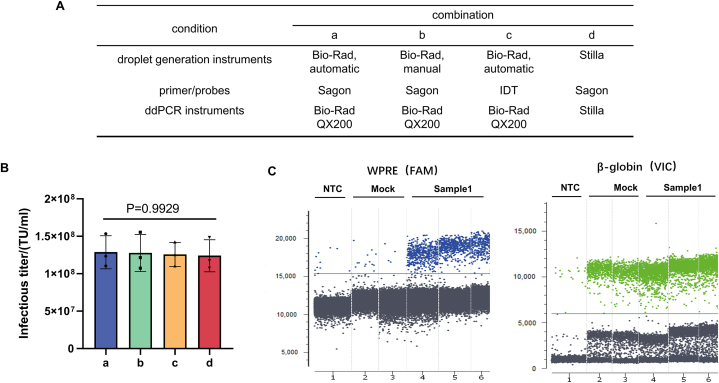
Validation of robustness (A) Multifactorial design showing conditions with different primer/probe sources and instruments. (B) Infectious titers of sample 1 under four different test conditions. One-way analysis of variance revealed a P-value of 0.9929 (P > 0.05), indicating no significant differences among the four conditions. (C) Droplet distribution under condition d.

To test the robustness of our method, we evaluated its performance with various equipment and reagents. For droplet generation, we used both the AutoDG Droplet Digital PCR System (automatic) and the QX200™ Droplet Generator (manual), with the Bio-Rad QX200 as the reading instrument in both cases. We tested two different ddPCR reading instruments: the QX200 (Bio-Rad) and the Naica (Stilla Technologies). Finally, we tested primers and probes from two synthesis companies: Sagon and IDT. We combined these factors with the four reaction conditions depicted in Fig. 7A, and tested nucleic acid extracts from HT1080 cells infected with sample 1. We observed no significant differences in detection results across the four conditions (Fig. 7B), indicating that our method demonstrates good robustness.

#### 2.2.6 Comparison of infectious titers determined by ddPCR and FACS

**Fig. 8:**
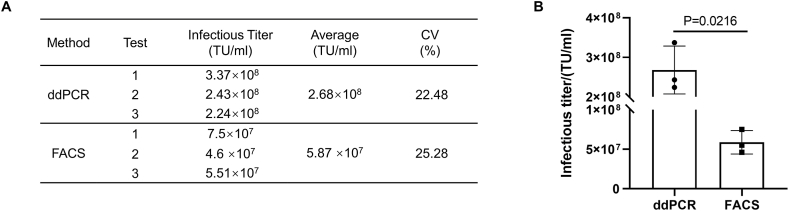
Comparison of infectious titers determined by ddPCR and FACS. (A) Lentiviral vector infectious titers were determined by three independent assays (both ddPCR and FACS). (B) The infectious titers determined by ddPCR and FACS significantly differed (P < 0.05). Three independent tests were performed for each method. P-values were determined by two-sample *t-tests*.

To compare the lentiviral vector titer determined by our ddPCR method with existing infectious titer measurement method FACS, we infected HT1080 cells with lentiviral vectors. After 3 days of incubation, the rate of CAR expression in the cells was detected using flow cytometry to calculate the viral infectious titer; the genomes of the remaining cells were extracted for ddPCR analysis. The infectious titer, based on ddPCR assessment, was approximately 4–5-fold higher than the titer determined by FACS (Fig. 8).

## 3 Discussion

This study established an efficient lentiviral vector infectious titer measurement method based on ddPCR targeting the *WPRE* and *β-globin* genes. We comprehensively validated the method's specificity, working range, LOQ, precision, accuracy, and robustness. The results demonstrated that our method is cost-effective, accurate, and reproducible. Compared with the method described in a previous study [7], our method has a shorter experimental period (culture for 3 days post-infection), which greatly improves detection efficiency.

It is important to note that the infectious titer of lentiviral vectors is determined under specific conditions using specific cell lines. Changes in target cell types and transduction conditions can significantly affect transduction efficiency [18]. The cell type used to quantify lentiviral vector infectious titer should meet certain criteria, such as susceptibility to infection, high repeatability, and stability. All of these factors contribute to methodological reliability and robustness. Both 293T cells and HT1080 cells are widely used in lentiviral vector research because of their susceptibility to infection [1,4,12,18]. However, in our study, HT1080 cells showed relatively higher infectious titers; this result was confirmed by FACS. Additionally, 293T cells tend to detach from culture dishes due to their poor adhesion to the wall, which can create difficulties in cell handling. Therefore, whereas 293T cells were used in the previous study [7], the present study utilized HT1080 cells to enhance sensitivity and stability.

To address the issue of residual plasmid DNA in lentiviral vector samples introduced during transient plasmid transfection, and its potential impact on infectious titer detection, two main approaches have been utilized: diluting or eliminating plasmid residues through multiple cell passages, or treating harvested cells with digestive enzymes to degrade residual plasmids. The first method is time-consuming, typically requiring 10–14 days of subculture that greatly reduce detection efficiency [7]. In this study, we treated harvested cells with digestive enzymes to eliminate the effect of residual plasmids on titer detection, thereby reducing the cell culture time to 3 days. We also examined the effects of different digestive enzymes. Currently, Benzonase and DNase I are commonly used in the quantification of lentiviral vector infectious titer [19,20]. Benzonase is a non-specific nuclease capable of degrading both DNA and RNA, whereas DNase I is a specific nuclease that primarily targets double-stranded DNA. We observed that the addition of either enzyme significantly reduced the infectious titer, indicating the presence of plasmid residues and the need for digestive enzyme treatment. Additionally, Benzonase treatment resulted in more complete plasmid digestion compared with DNase I treatment. To demonstrate that Benzonase treatment can eliminate all non-transduced plasmid, we created an artificial positive control sample consisting of a mixture of lentiviral plasmid and HT1080 cells. The VCN measured for this control sample decreased to zero after Benzonase treatment, demonstrating complete plasmid digestion.

Another highlight of this study is its systematic methodological validation, which encompassed the entire experimental process from cell culture to ddPCR. Specificity validation results showed that the method accurately identified and eliminated interference from other related viruses or non-target substances. The addition of nevirapine, which blocks lentiviral vector transduction, further confirmed the methodological specificity. The working range of the method is approximately 3100 to 1.55E6 TU/ml, suggesting a wide range of applications, including the detection of samples with relatively low titers (around 3100 TU/ml). Importantly, samples with high titers (above 10^7^ TU/ml) must be diluted to this range for accurate results. The evaluation of precision demonstrated that our method exhibits good repeatability, with a CV ≤ 25 %. This finding is consistent with the high reproducibility of the ddPCR method reported in the literature [21]. Owing to the lack of a recognized lentiviral vector reference for overall accuracy verification concerning infectious titer quantification, we chose a cell line with known gene copy numbers to validate the accuracy of the ddPCR method itself. The accuracy of our ddPCR method exceeded 90 %, which is comparable to the accuracy of the ddPCR method established by Wang et al. [7]. The robustness of the method was validated by introducing subtle but deliberate changes to method variables and assessing their impacts on method performance [22], thus facilitating wider applicability. In this study, we explored variations between automated and manual droplet generation, as well as different types of ddPCR detection instruments and synthetic primer manufacturers. Our ddPCR-based method remained robust under all tested conditions, indicating that it is reliable and reproducible.

Finally, in the comparison between ddPCR and FACS, infectious titers determined by ddPCR were approximately 4–5-fold higher than those measured by FACS. The lower infectious titers according to FACS may be related to two factors mentioned in the Introduction. First, FACS cannot distinguish between cells infected by a single vector and those infected by multiple vectors. Second, FACS cannot detect integrated but non-functional viral vector copies that do not lead to transgene expression [5,6]. However, we also acknowledge that the ddPCR method established by Wang et al. displays good comparability with FACS. This comparability may exist because the lentiviral vector used is labeled with GFP, and thus the target protein GFP can be detected if the vector is expressed; cell surface expression is not required. Furthermore, GFP detection does not rely on antibody binding, whereas the use of different types of antibodies (e.g., antibodies against scFv or Protein L) in flow cytometry may affect the results.

## 4 Conclusion

We have established a ddPCR method for lentiviral vector infectious titer detection by targeting *WPRE* and *β-globin*, which exhibits good specificity, repeatability, accuracy, and robustness. This method could be utilized in the development and manufacturing of cell therapy products.

## 5 Materials & methods

### 5.1 Cells and viruses

HT1080 cells and 293T cells were purchased from ATCC in 2018 and 2014, respectively. STR identification and mycoplasma testing were performed to confirm cell line identity and the absence of mycoplasma contamination. Both HT1080 and 293T cells were cultured in MEM medium supplemented with 10 % fetal bovine serum. Five lentiviral vector samples from different sources were designated as samples 1–5. Murine leukemia virus (MuLV) and gibbon ape leukemia virus (GALV) were maintained in our laboratory.

### 5.2 Primers and probes

The primer and probe sequences for the detection of *WPRE* and *β-globin* used in ddPCR are listed below. Gene Runner software was used to design primers and probes. For the *WPRE* primers and probe, we used the reference sequence with GenBank number FJ797421.1; the amplicon is located at positions 3896–4037 bp, with a length of 142 bp. For the *β-globin* primers and probe, we used the reference sequence with GenBank number AH001475.2; the amplicon is located at positions 1575–1681, with a length of 107 bp. These constructs were synthesized by Sagon and IDT. Primer and probe sequences for *LTR* gene detection are not publicly available because they were developed by a CAR-T production company.

*WPRE*-FP: 5′-CATTGCCACCACCTGTCA-3’;

*WPRE*-RP: 5′-CGACAACACCACGGAATT-3’;

*WPRE*-probe: 5′FAM-AGGCAGGCGGCGATGAGT-3′BHQ.

*β-globin*-FP: 5′-ACACAACTGTGTTCACTAGCAA-3’;

*β-globin*-RP: 5′-CTTCATCCACGTTCACCTTG-3’;

*β-globin*-probe: 5′VIC-CATGGTGCACCTGACTCCTGAG-3′BHQ.

### 5.3 Optimization of experimental parameters

In the lentiviral vector titer detection method, the following key experimental parameters were optimized: (1) Cell line selection: HT1080 or 293T cells were used as candidate cell lines. HT1080 cells and 293T cells were adjusted to 1.5 × 10^5^ cells/ml and 2 × 10^5^ cells/ml [23], respectively. The diluted cells were then seeded into 6-well plates at a volume of 2 ml per well and incubated at 37 °C with 5 % CO₂ for 24 h. HT1080 and 293T cells were transduced with lentiviral vector at dilutions of 1:1000 and 1:10000, then cultured for 3 days. Next, the cells were treated with 100 U/ml Benzonase for 1.5 h. Cell genomic DNA was extracted and detected by ddPCR. (2) Target gene selection: The *WPRE* or *LTR* gene was used as the target gene. HT1080 cells transduced with lentivirus vector (diluted 1000-fold and 10000-fold) were treated with Benzonase, and genomic DNA was extracted. ddPCR was then performed targeting the *WPRE* or *LTR* gene. (3) Enzyme treatment: During cell harvesting, we tested different types of enzymes (Benzonase and DNase I) for cell treatment and residual plasmid digestion to ensure more accurate results. Experimental conditions included 10 U/ml for 0.5 h, 10 U/ml for 2.5 h, 100 U/ml for 0.5 h, 100 U/ml for 2.5 h, and 55 U/ml for 1.5 h. Genomic DNA from these treated cells was then extracted for ddPCR detection and infectious titer calculation. (4) Optimal enzyme concentration and digestion interval: The enzyme concentration and digestion interval were optimized based on factors such as digestion efficiency, operation time, and cost.

### 5.4 Method validation

Method validation included the following parameters: (1) Specificity: Specificity refers to the ability of the analytical method to accurately and selectively identify the target analyte in the presence of other components in the sample. The positive control samples included five routine lentiviral vector samples, designated samples 1–5. The negative control samples consisted of a blank matrix of 293T cell lysate and two non-related viruses, MuLV and GALV. Both positive and negative samples were used for infection experiments, and genomic DNA was extracted for ddPCR detection to evaluate methodological specificity. Additionally, nevirapine, a reverse transcriptase inhibitor when used at a final concentration of 10 μmol/L [15], was utilized to confirm that the measured vector DNA titer was transduction-specific and not due to pseudotransduction by residual plasmid DNA present in lentiviral vector preparations from transient transfection systems. (2) Working range and LOQ: HT1080 cells were infected with lentiviral vectors at different dilutions, with three transduction replicates for each dilution and three ddPCR replicates for each transduction. The LOQ and the working range of the method were assessed by the acceptability of CV. (3) Precision: The precision of the method was determined by evaluating intra-assay and inter-assay variation. Intra-assay repeatability involved conducting three transduction replicates in one infection assay using sample 1 to transduce HT1080 cells; three ddPCR replicates were performed for each transduction. Inter-assay repeatability was assessed four times on different days by two operators. (4) Accuracy: The accuracy experiment used the ddPCR method to detect the VCN of Jurkat-CAR cells with known copy numbers. (5) Robustness: The robustness of the method was investigated by analyzing manual and automatic droplet generation instruments from Bio-Rad, different brands of ddPCR instruments (QX200 Bio-Rad and Naica Stilla Technologies), and primers synthesized by different suppliers (Sagon and IDT).

### 5.5 FACS

Three days post-infection, cells were trypsinized and washed. Next, 1E6 cells were stained with Rabbit Anti-Mouse FCM63 scFv Monoclonal Antibody (BioSwan Laboratories, Shanghai, China) at room temperature for 15 min. The cells were subsequently washed and resuspended in phosphate-buffered saline, then analyzed using a Fortessa Flow Cytometer (BD). The phycoerythrin（PE）channel was selected for detection, and uninfected cells served as a negative control. Only viral dilutions that produced 5%–20 % CAR-positive cells were used to calculate infectious titer, based on the following formula: Infectious titer (TU/ml) = (% of CAR-positive cells × number of transduced cells per well)/(virus volume (ml)) × dilution factor.

## Ethics approval

This study did not involve human subjects or animal experiments.

## Data availability statement

All data generated or analyzed during this study are included in this published article. The original data for Fig. 1, Fig. 2, Fig. 3, Fig. 5, Fig. 7B, and 8B have been deposited in Mendeley Data: https://data.mendeley.com/drafts/2ds7jvvvg7.

## CRediT authorship contribution statement

**Xueling Wu:** Writing – review & editing, Supervision, Project administration, Methodology, Formal analysis, Conceptualization. **Xiaoya Zhou:** Writing – review & editing, Writing – original draft, Visualization, Validation, Formal analysis, Data curation, Conceptualization. **Yueming Wang:** Validation, Software, Methodology. **Jian Wu:** Visualization, Validation, Conceptualization. **Qian Liang:** Resources. **Xu Yang:** Writing – review & editing, Conceptualization. **Kehua Zhang:** Software, Project administration, Methodology, Funding acquisition, Formal analysis, Conceptualization. **Shufang Meng:** Supervision, Resources, Project administration, Methodology, Investigation, Funding acquisition, Conceptualization.

## Declaration of competing interest

The authors declare no competing financial interests or personal relationships that could have influenced the work reported in this paper.
